# Supplementary material for: Antioxidant Potential of the Sweet Whey-Based Beverage Colada after the Digestive Process and Relationships with the Lipid and Protein Fractions
Source: Antioxidants (Basel). 2022 Sep 16;11(9):1827. doi: 10.3390/antiox11091827 (PMC9495724; doi:10.3390/antiox11091827)
Supplement: Supplementary file 1 [file antioxidants-11-01827-s001.zip › antioxidants-1880194-supplementary.pdf]

TABLES SENSORY EVALUATION

Table S1. Discriminant sensory analysis of different formulations of *Colada* (25-5 % Maracuyá juice) by trained judges (n=5)[1].

Categories	Attributes	25%		15%[3]		10%		7%		5%	
		Mean	CVC[2]	Mean	CVC	Mean	CVC	Mean	CVC	Mean	CVC
Flavor	Bitter	3	0.56032	2	0.48032	3	0.64032	2	0.36032	2	0.32032
	Salty	2	0.48032	2	0.36032	3	0.60032	2	0.48032	2	0.40032
	**Acidic**	**4**	**0.76032**	**5**	**0.92032**	**3**	**0.68032**	**3**	**0.60032**	**3**	**0.56032**
	**Sweet**	**4**	**0.76032**	**5**	**0.96032**	**3**	**0.68032**	**3**	**0.64032**	**3**	**0.64032**
	Umami	2	0.40032	2	0.32032	3	0.64032	2	0.40032	2	0.36032
	Spicy	3	0.56032	3	0.68032	2	0.48032	2	0.40032	2	0.36032
	Astringent	2	0.36032	2	0.48032	2	0.40032	2	0.32032	2	0.36032
Color	Brightness	2	0.40032	3	0.56032	2	0.32032	2	0.40032	2	0.40032
	Transparency	3	0.56032	2	0.44032	2	0.48032	2	0.48032	2	0.36032
	**Opacity**	**4**	**0.80032**	**5**	**0.92032**	**4**	**0.88032**	**4**	**0.80032**	**4**	**0.80032**
	**Pallor**	**4**	**0.88032**	**5**	**0.96032**	**5**	**0.92032**	**4**	**0.84032**	**4**	**0.88032**
Texture	Pasty	3	0.60032	3	0.64032	3	0.60032	2	0.36032	2	0.40032
	**Tender**	**4**	**0.84032**	**5**	**0.96032**	**4**	**0.88032**	**4**	**0.88032**	**4**	**0.80032**
	Rubbery	3	0.68032	2	0.32032	3	0.60032	1	0.28032	2	0.32032
	Fluid	3	0.64032	2	0.48032	2	0.44032	2	0.44032	1	0.28032
	**Dense**	**4**	**0.84032**	**5**	**0.96032**	**5**	**0.92032**	**4**	**0.88032**	**4**	**0.88032**
	**Viscous**	**4**	**0.80032**	**5**	**0.92032**	**4**	**0.88032**	**4**	**0.88032**	**4**	**0.80032**
	Elastic	2	0.48032	3	0.64032	3	0.60032	2	0.44032	3	0.52032
	Sticky	2	0.40032	1	0.28032	3	0.60032	2	0.44032	2	0.36032
	Adherent	3	0.52032	3	0.52032	2	0.48032	2	0.44032	2	0.36032
	**Fine**	**4**	**0.72032**	**5**	**0.92032**	**4**	**0.80032**	**4**	**0.88032**	**4**	**0.80032**
	Liquid	3	0.56032	3	0.64032	3	0.60032	3	0.56032	2	0.36032
General Appearance	**Low Dense**	**4**	**0.88032**	**5**	**0.96032**	**4**	**0.80032**	**4**	**0.84032**	**4**	**0.84032**
	Dense	2	0.48032	3	0.56032	3	0.60032	2	0.32032	2	0.40032
	Very Dense	3	0.52032	2	0.44032	3	0.56032	2	0.40032	2	0.44032

1 The measure scale was of five points (1: not perceived, 2: poorly perceived, 3: fairly perceived, 4: well perceived and 5: excellent perceived)

2 CVC: Concordance Validity Coefficient (from < 0.60, very deficient, to > 0.90, excellent)

3 The *Colada* at 15% Maracuyá juice obtained the best score with CVC values of 0.92 - 0.96.

Table S2. Hedonic evaluation of *Colada* (15 % Maracuyá juice) by consumers (n=80)[1].

Categories	Attributes	Mean	CVC[2]
Flavor	Bitter	1	0.29
	Salty	3	0.58
	**Acidic**	**4**	**0.85**
	**Sweet**	**4**	**0.87**
	Umami	2	0.48
	Spicy	3	0.50
	Astringent	2	0.50
Color	Brightness	3	0.52
	Transparency	2	0.44
	**Opacity**	**4**	**0.80**
	**Pallor**	**4**	**0.82**
Texture	Pasty	1	0.30
	**Tender**	**4**	**0.90**
	Rubbery	1	0.30
	Fluid	3	0.65
	**Dense**	**4**	**0.80**
	**Viscous**	**4**	**0.85**
	Elastic	1	0.30
	Sticky	3	0.65
	Adherent	1	0.30
	**Fine**	**4**	**0.80**
	Liquid	1	0.30
General Appearance	**Low Dense**	**4**	**0.83**
	Dense	1	0.30
	Very Dense	1	0.30

[1] The measure scale was of five points (1: not perceived, 2: poorly perceived, 3: fairly perceived, 4: well perceived and 5: excellent perceived).

[2] CVC: Concordance Validity Coefficient (from < 0.60, very deficient, to > 0.90, excellent). Consumers evaluated the beverage with CVC between 0.80 and 0.90.

FIGURES CHOROMATOGRAMS

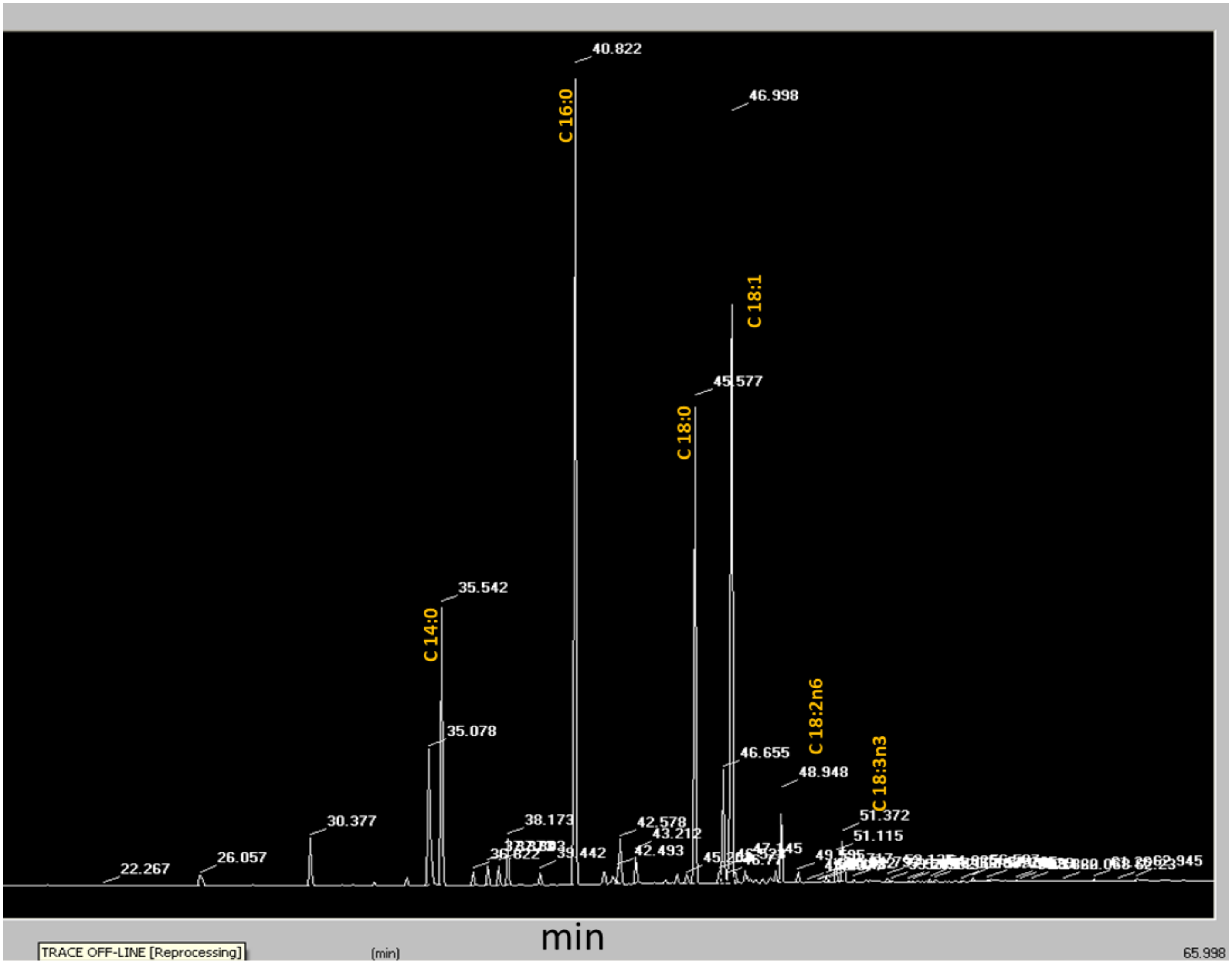

Figure S1. Chromatogram (GC) of fatty acid methyl esters (FAME) of the whey-beverage (*Colada*) before the in vitro digestion.

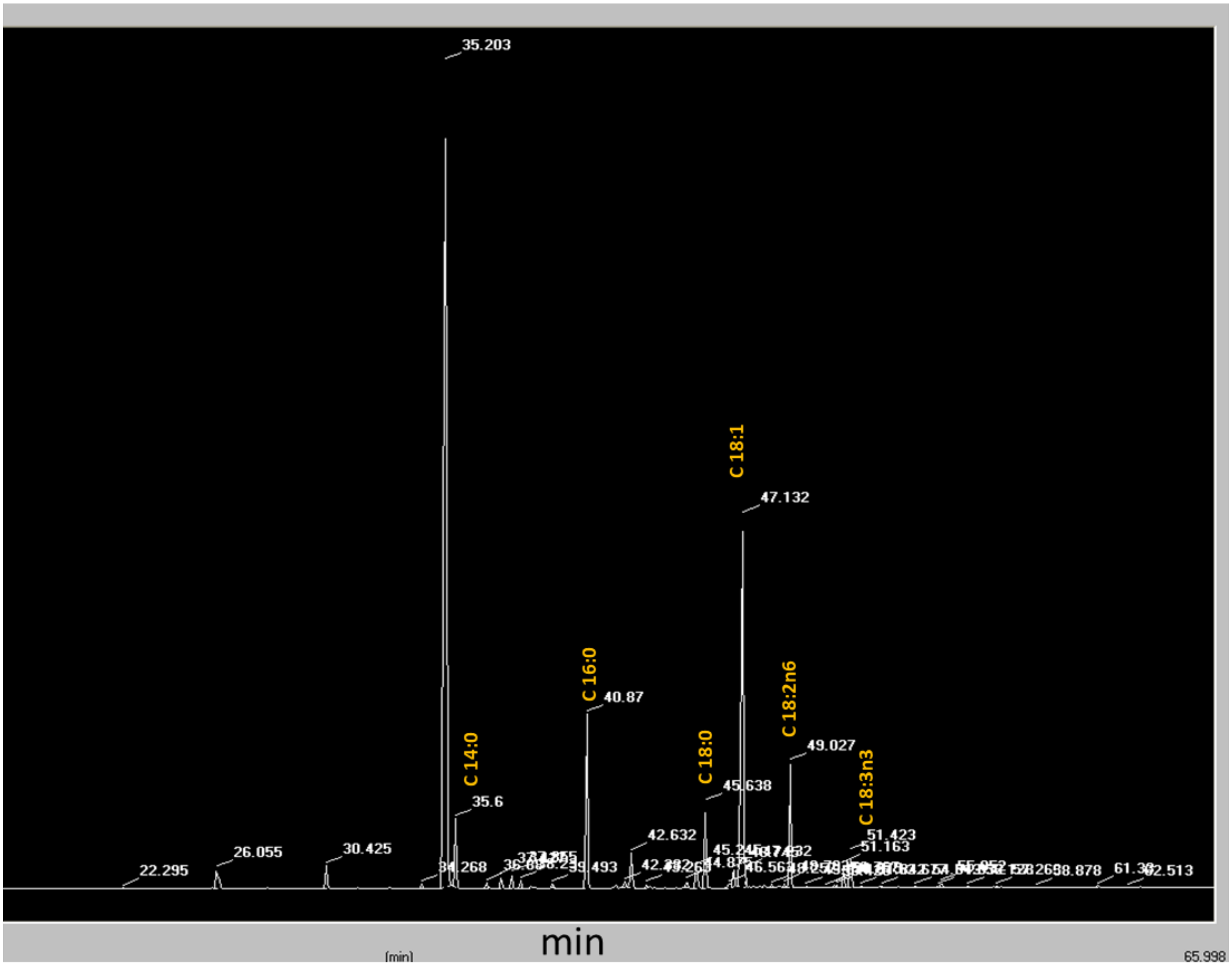

Figure S2. Chromatogram (GC) of fatty acid methyl esters (FAME) of the whey-beverage (*Colada*) after the in vitro digestion (bioaccessible fraction).

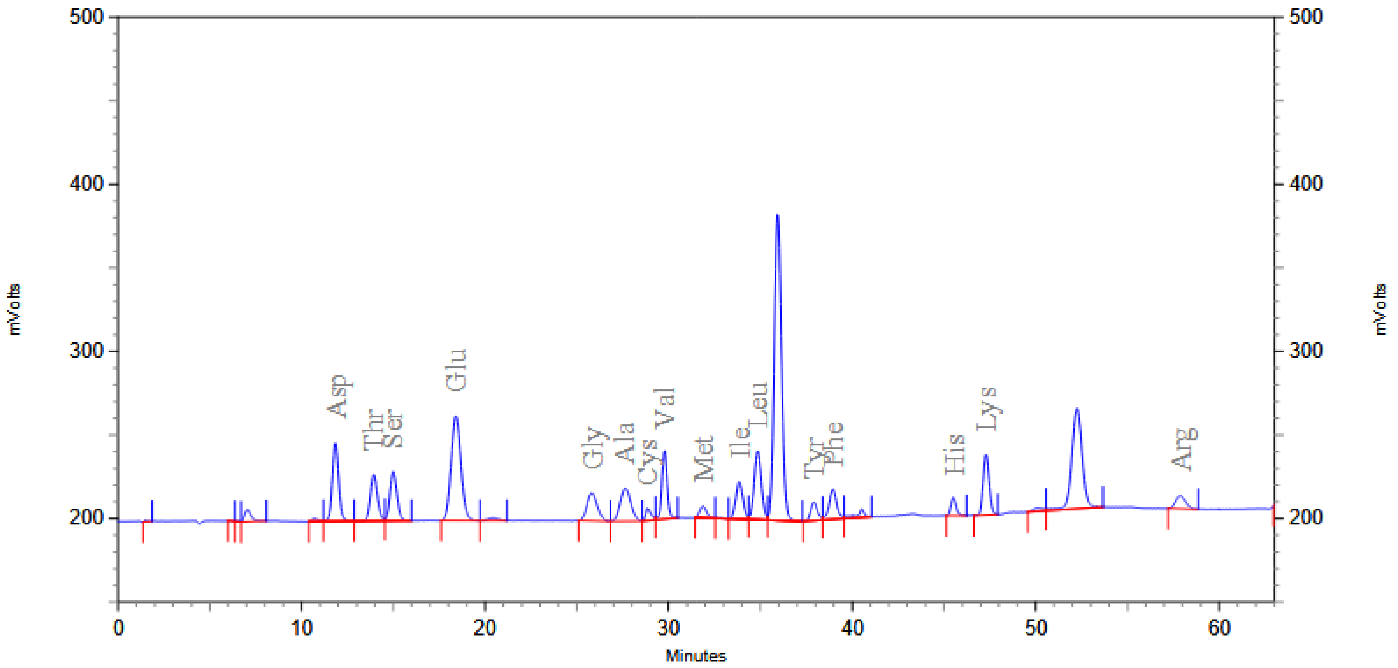

Figure S3. Chromatogram (HPLC) of amino acids of the whey-beverage (*Colada*) (acid hydrolysis).

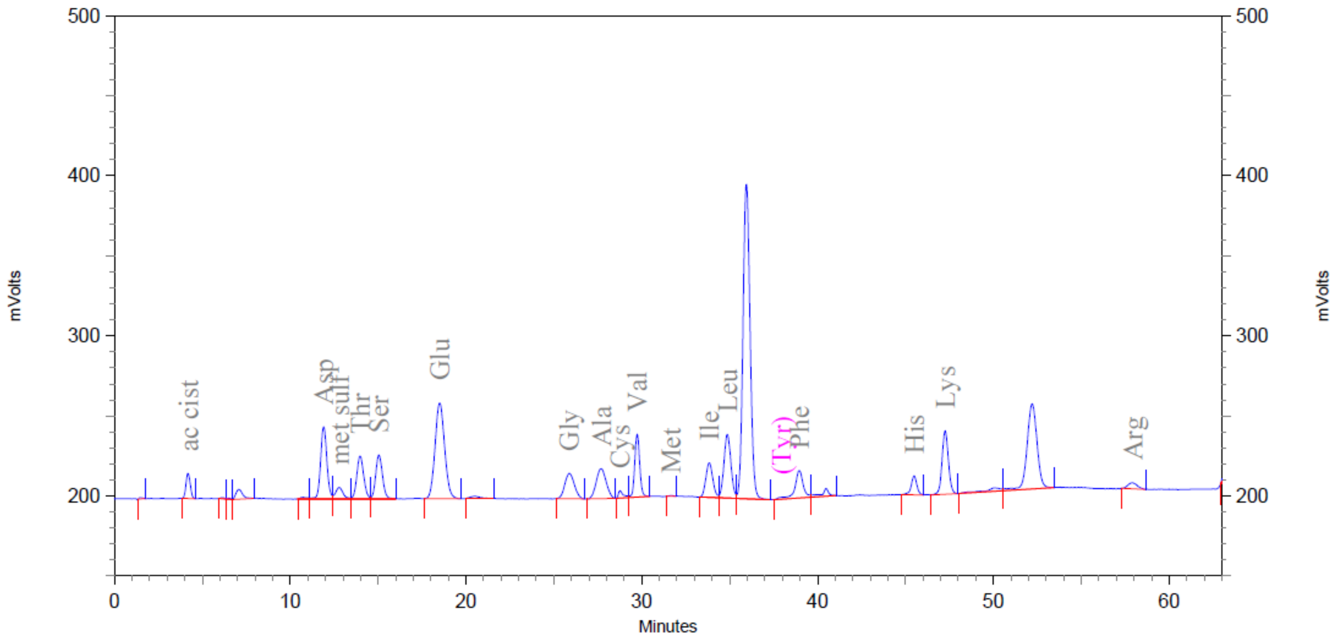

Figure S4. Chromatogram (HPLC) of amino acids of the whey-beverage (*Colada*) (performic oxidation).
